# Terahertz Broadband Polarization Conversion for Transmitted Waves Based on Graphene Plasmon Resonances

**DOI:** 10.3390/nano11010056

**Published:** 2020-12-28

**Authors:** Anqi Yu, Dahai Yu, Zhenyu Yang, Xuguang Guo, Yuxiang Ren, Xiaofei Zang, Alexei V. Balakin, Alexander P. Shkurinov, YiMing Zhu

**Affiliations:** 1 Shanghai Key Lab of Modern Optical System, Terahertz Technology Innovation Research Institute, Terahertz Spectrum and Imaging Technology Cooperative Innovation Center, University of Shanghai for Science and Technology, 516 Jungong Road, Shanghai 200093, China; yuanqi@mail.sitp.ac.cn (A.Y.); 192380304@st.usst.edu.cn (Z.Y.); xgguo@usst.edu.cn (X.G.); 182390295@st.usst.edu.cn (Y.R.); xfzang@usst.edu.cn (X.Z.); a.v.balakin@physics.msu.ru (A.V.B.); ashkurinov@physics.msu.ru (A.P.S.); 2 Shanghai Institute of Intelligent Science and Technology, Tongji University, Shanghai 200092, China; 3 Focused Photonics (Hangzhou) Inc., No: 760, Bin’an Road, Binjiang District, Hangzhou 310052, China; dahai_yu@fpi-inc.com; 4 Faculty of Physics and International laser Center, Lomonosov Moscow State University, Leninskie Gory 1-2, 19991 Moscow, Russia; 5 ILIT RAS–Branch of the FSRC “Crystallography and Photonics” RAS, Svyatoozerskaya 1, 140700 Shatura, Moscow Region, Russia

**Keywords:** terahertz, graphene, plasmons, polarization conversion

## Abstract

We applied the harmonic oscillator model combined with the transfer matrix method to study the polarization conversion for transmitted waves in metallic grating/plasmon-excitation layer/metallic grating structure in the terahertz (THz) region. By comparing the calculated spectra and the simulated (by the finite-difference-time-domain method) ones, we found that they correspond well with each other. Both methods show that the Drude background absorption and the excited plasmon resonances are responsible for polarization conversion. The transmission is close to 0 when the distance between the top/bottom metallic gratings and gated graphene is an integer multiple of half the wavelength of the incident wave (in the dielectrics), at which points the plasmon resonances are greatly suppressed by the destructive interference between the backward/forward electromagnetic waves and that reflected by the top/bottom metallic gratings. Away from these points, the transmission can be higher than 80%. The electron density and the excitation efficiency of the plasmon-excitation layer were found to be important for the bandwidth of the polarization conversion window, while the scattering rate was found to influence mainly the polarization conversion rate. Multi-broadband polarization conversion is realized by exciting plasmon modes between the 0 transmission points in the THz region.

## 1. Introduction

Polarization rotators are basic elements for THz applications because polarization is one of the fundamental properties that conveys valuable information of electromagnetic waves. Conventional approaches use birefringence [1,2], total internal reflection effects [3,4], and the Faraday effect [5,6,7] to change the polarization of electromagnetic waves. However, birefringence rotators rely on the accumulation of the difference in phase, such that precise control of thickness is required, and these rotators are usually narrow-band and bulky. Total internal reflection rotators rely on multiple total reflections, such that they require complex design and fabrication. Faraday rotators need the application of an external magnetic field on magneto-optical materials, resulting in high cost and fabrication difficulties.

In recent years, metamaterials have been demonstrated to be effective in rotating the polarization of the incident waves. Transmission-type quarter wave plates [8,9,10,11,12,13,14], half wave plates [12,13,14,15], and polarization rotators [16,17,18,19,20,21,22,23] have been realized by dielectric or metallic metamaterials, both theoretically and experimentally. However, with only one layer of metamaterials, the polarization conversion is usually of a comparatively narrow relative bandwidth (<30%) [9,10,11,12,13,14] or low efficiency [8]. Since the proposal of a Fabry–Pérot-like cavity formed by two orthogonal sets of metallic gratings by Grady et al. [16,17,18,23], structures with two or more layers, which consist of at least one set of bottom metallic gratings, show both a comparatively broad relative bandwidth (>50%) and a high polarization conversion rate (PCR). The bottom gratings can reflect the transmitted light with parallel polarization, and then the reflected light can interact with the metamaterial again, and part of the reflected light will be transferred to the desired polarization and pass through the bottom gratings, thus enhancing both relative bandwidth and PCR. Similarly, plasmons can also be used to change the polarization of the incidence. Once the incidence passes through the plasmon-excitation layer, both the phase and the amplitude will be changed by the plasmon resonances, and then the polarization will be changed accordingly. Recently, Zhao et al. proposed to encapsulate two periodic graphene ribbons in between two sets of metallic gratings and realized broadband THz polarization conversion in the THz frequencies [24]. However, up to now, a detailed model description of both relative bandwidth and PCR was still lacking.

In this work, we apply the transfer matrix method (TMM) to study the polarization conversion of the metallic grating/plasmon-excitation layer/metallic grating structure. The plasmon-excitation layer is described by the harmonic oscillator model. Cross-polarization conversion in such structures is described by using the proposed method. The polarization conversion in such a structure is also simulated by using the finite-difference-time-domain (FDTD) method. The spectra of both methods correspond well with each other, which means that the model offers quick and effective prediction for polarization converters with similar sandwich structures. The model shows that the polarization conversion is influenced by the modulation in amplitude and phase caused by both the plasmon resonances and Drude background absorption. We find that both the duty cycle of the plasmon-excitation region and the Fermi level of graphene are crucial for the relative bandwidth, while the carrier scattering rate (or the relaxation time) will influence the PCR. The distance between the plasmon-excitation layer and the top/bottom metallic gratings will also influence the PCR. Based on these analyses, multi-broadband polarization conversion is realized by taking gated graphene as the plasmon-excitation layer. Such a multi-broadband polarization conversion can be actively tuned by changing the Fermi energy of the graphene. This study is helpful for the design of polarization rotators with similar sandwich structures, and it is beneficial for THz manipulation applications.

## 2. Model Descriptions

**Figure 1:**
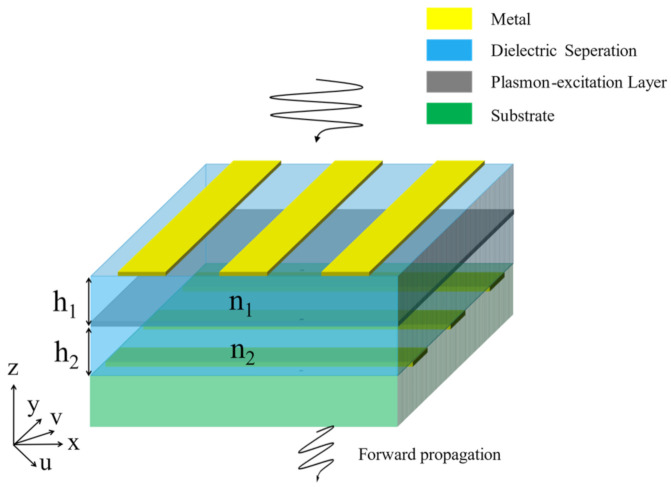
The studied metallic gratings/plasmon-excitation layer/metallic gratings structure.

Figure 1 schematically shows the studied structure, which consists of the top metallic gratings, an upper dielectric separation (separating the top gratings and the plasmon-excitation layer), the plasmon-excitation layer, a lower dielectric separation (separating the plasmon-excitation layer and the bottom gratings), the bottom metallic gratings, and the substrate. Since x-polarized waves only are allowed to pass through the top gratings and y-polarized waves only are allowed to pass through the bottom gratings, PCR is directly defined as the transmission. The top metallic gratings allow the propagation of x-polarized incidence, while the bottom metallic gratings allow the propagation of y-polarized incidence. It should be noted that the gray layer just indicates the position of the plasmon-excitation layer instead of indicating a complete sheet of a certain material.

The gray layer has possible plasmon excitations in the u coordinate and v coordinate, with $\vec{u}=\frac{1}{\sqrt{2}}(\vec{x}-\vec{y})$ and $\vec{v}=\frac{1}{\sqrt{2}}(\vec{x}+\vec{y})$. Here, the u and v coordinates are 45° to the *x* and *y* coordinates, because 45° is usually the optimal choice in this scenario. The boundary matrices are described by 4 × 1 matrices (two polarizations times two directions), and the transfer matrices are described by 4 × 4 matrices. At the very beginning, the boundary matrix of the electromagnetic waves at the lower surface of the bottom gratings is:

(1) $$\left(\begin{array}{l} E_{x}^{+}\\ E_{x}^{-}\\ E_{y}^{+}\\ E_{y}^{-} \end{array}\right)=\left(\begin{array}{l} 0\\ 0\\ 1\\ 0 \end{array}\right)$$

where the subscripts “*x*” and “*y*” indicate the polarizations, and the superscripts “+” and “−” indicate forward-propagation (−*z* direction) and backward-propagation (+*z* direction), respectively. In the simulation, the widths of the metallic gratings are 1 μm and the period is 1.414 μm. Although not shown here, the transmission is above 99.7% through a suspended metallic grating layer for the THz incidence polarized perpendicular to the gratings. For the THz incidence polarized parallel to the gratings, the transmission is 0%. Therefore, the gratings are simply considered transparent for perpendicularly polarized waves and mirror for parallel-polarized waves, and then, the boundary matrix at the upper surface of the bottom grating is written as:

(2) $$T_{bottom}=\left(\begin{array}{l} E_{x}^{+}\\ E_{x}^{-}\\ E_{y}^{+}\\ E_{y}^{-} \end{array}\right)=\left(\begin{array}{l} E_{x0}\\ -E_{x0}\\ 1\\ 0 \end{array}\right)$$

where *E_x_*_0_ indicates the electric field of the x-component of the forward-propagating wave arriving at the upper surface of the bottom gratings, and its value will be given later by boundary conditions. Here, the phase change of *E_y_*^+^ through the bottom gratings are neglected because of the negligible thickness of the metallic gratings (100 nm) compared with the wavelength of the incidence. The transfer matrices for the upper and lower dielectric separations are:

(3) $$M_{upper/lower}=\left(\begin{array}{cccc} e^{-ik_{0}n_{i}h_{i}} & 0 & 0 & 0\\ 0 & e^{ik_{0}n_{i}h_{i}} & 0 & 0\\ 0 & 0 & e^{-ik_{0}n_{i}h_{i}} & 0\\ 0 & 0 & 0 & e^{ik_{0}n_{i}h_{i}} \end{array}\right)$$

where *k*_0_ is the wavevector in the vacuum, and *n_i_* and *h_i_* are the refractive indices and thicknesses of the upper (*i* = 1) and lower (*i* = 2) dielectric separations, respectively. Here, the expression of forward-propagation is defined as *e*^−*i*(*kz* − *ωt*)^, and then, the minus signs are given to the exponents of the diagonal elements that describe the forward-propagation. At the plasmon-excitation layer, the transfer matrix in the *u*-*v* coordinates reads [25,26]:

(4) $$M_{res}=\frac{1}{2}\left(\begin{array}{cccc} 1+\frac{n_{2}}{n_{1}}+\frac{\sigma_{u}\left(\omega \right)}{\varepsilon_{0}c} & 1-\frac{n_{2}}{n_{1}}+\frac{\sigma_{u}\left(\omega \right)}{\varepsilon_{0}c} & 0 & 0\\ 1-\frac{n_{2}}{n_{1}}-\frac{\sigma_{u}\left(\omega \right)}{\varepsilon_{0}c} & 1+\frac{n_{2}}{n_{1}}-\frac{\sigma_{u}\left(\omega \right)}{\varepsilon_{0}c} & 0 & 0\\ 0 & 0 & 1+\frac{n_{2}}{n_{1}}+\frac{\sigma_{v}\left(\omega \right)}{\varepsilon_{0}c} & 1-\frac{n_{2}}{n_{1}}+\frac{\sigma_{v}\left(\omega \right)}{\varepsilon_{0}c}\\ 0 & 0 & 1+\frac{n_{2}}{n_{1}}-\frac{\sigma_{v}\left(\omega \right)}{\varepsilon_{0}c} & 1+\frac{n_{2}}{n_{1}}-\frac{\sigma_{v}\left(\omega \right)}{\varepsilon_{0}c} \end{array}\right)$$

accompanied with:

(5) $$M_{xy\to uv}=\frac{1}{\sqrt{2}}\left(\begin{array}{cccc} 1 & 0 & -1 & 0\\ 0 & 1 & 0 & -1\\ 1 & 0 & 1 & 0\\ 0 & 1 & 0 & 1 \end{array}\right)$$

(6) $$M_{uv\to xy}=\frac{1}{\sqrt{2}}\left(\begin{array}{cccc} 1 & 0 & 1 & 0\\ 0 & 1 & 0 & 1\\ -1 & 0 & 1 & 0\\ 0 & -1 & 0 & 1 \end{array}\right)$$

with *σ_u_*(*ω*) and *σ_v_*(*ω*) being the effective conductivities in the *u* and *v* coordinates, respectively. *ε*_0_ is the vacuum permittivity and *c* is the speed of light in vacuum. Equations (5) and (6) are the coordinate transformation matrices from *x*-*y* to *u*-*v* and the reverse, respectively. Then, the transfer matrix at the lower surface of the top gratings is:

(7) $$\left(\begin{array}{l} E_{x}^{+}\\ E_{x}^{-}\\ E_{y}^{+}\\ E_{y}^{-} \end{array}\right)=\left(\begin{array}{c} e^{-ik_{0}(n_{1}h_{1}+n_{2}h_{2})}E_{x0}+e^{-ik_{0}n_{1}h_{1}}A+e^{-ik_{0}n_{1}h_{1}}B\\ -e^{ik_{0}(n_{1}h_{1}+n_{2}h_{2})}E_{x0}-e^{ik_{0}n_{1}h_{1}}A-e^{ik_{0}n_{1}h_{1}}B\\ e^{-ik_{0}(n_{1}h_{1}+n_{2}h_{2})}-e^{-ik_{0}n_{1}h_{1}}A+e^{-ik_{0}n_{1}h_{1}}B\\ e^{ik_{0}n_{1}h_{1}}A-e^{ik_{0}n_{1}h_{1}}B \end{array}\right)$$

(8) $$A=\frac{\sigma_{u}\left(\omega \right)}{4\varepsilon_{0}c}\left[-2i\times \sin\left(k_{0}n_{2}h_{2}\right)E_{x0}-e^{-ik_{0}n_{2}h_{2}}\right]$$

(9) $$B=\frac{\sigma_{v}\left(\omega \right)}{4\varepsilon_{0}c}\left[-2i\times \sin\left(k_{0}n_{2}h_{2}\right)E_{x0}+e^{-ik_{0}n_{2}h_{2}}\right]$$

At the lower surface of the top gratings, $E_{y}^{+}=-E_{y}^{-}$ should be satisfied, which requires:

(10) $$E_{x0}=\frac{\frac{e^{-ik_{0}\left(n_{1}h_{1}+n_{2}h_{2}\right)}}{-2i\times \sin\left(k_{0}n_{1}h_{1}\right)}\times 4\varepsilon_{0}c+\left[\sigma_{u}\left(\omega \right)+\sigma_{v}\left(\omega \right)\right]e^{-ik_{0}n_{2}h_{2}}}{\left[\sigma_{u}\left(\omega \right)-\sigma_{v}\left(\omega \right)\right]\left(-2\sin\left(k_{0}n_{2}h_{2}\right)\right)}$$

Since more than 99.7% incidence can pass through the top gratings, the transfer matrix of the top gratings is:

(11) $$M_{TG}=\frac{1}{2}\left(\begin{array}{cccc} 1+n_{1} & 1-n_{1} & 0 & 0\\ 1-n_{1} & 1+n_{1} & 0 & 0\\ 0 & 0 & 0 & 0\\ 0 & 0 & 0 & 0 \end{array}\right)$$

Finally, we have:

(12) $$T_{top}=\left(\begin{array}{l} E_{inc}\\ E_{ref}\\ 0\\ 0 \end{array}\right)=M_{TG}M_{upper}M_{uv\to xy}M_{res}M_{xy\to uv}M_{lower}T_{bottom}$$

where |*E_inc_*|^2^ and |*E_ref_*|^2^ represent the total incidence and reflection, respectively. Note that the total transmitted electric field as given in Equation (1) has been normalized to *E_x_*^2^ + *E_y_*^2^ = 1, and then the transmission is expressed as:

(13) $$T={\left|\frac{1}{E_{inc}}\right|}^{2}$$

Obviously, if there are resonances with the same frequency and amplitude in both u and v coordinates, the denominator of *E_x0_* is 0, and then *E_x0_* is infinite, resulting in infinite *E_inc_*. As a result, the transmission is 0%. Physically, it can be understood as follows: plasmons excited at both coordinates have an equal impact on the phase and amplitude of *E_u_* and *E_v_*, so there is no anisotropy and the polarization is not rotated at all. Consequently, the x-polarized incidence will be completely reflected back by the bottom gratings. In order to effectively change the polarization of the incidence, we assume that there are plasmon resonances only in the u coordinate. For simplicity, the refractive index of the substrate and those of the two dielectric separations are assumed to be the same, with *n* = *n*1 = *n*2. Then, *E_inc_* can be simplified as:

(14) $$\begin{array}{c} E_{inc}=\frac{\left(n+1\right)}{2}e^{-ik_{0}n\left(h_{1}+h_{2}\right)}\left\{\frac{\left(1-n\right)e^{ik_{0}n\left(h_{1}+h_{2}\right)}-\left(1+n\right)e^{-ik_{0}n\left(h_{1}+h_{2}\right)}}{4\left(n+1\right)\sin\left(k_{0}nh_{1}\right)\sin\left(k_{0}nh_{2}\right)}\frac{4\varepsilon_{0}c}{\sigma_{u}\left(\omega \right)}\right.\\ \left.+\left[\left(n-1\right)e^{2ik_{0}nh_{1}}+\left(n+1\right)\right]\frac{e^{ik_{0}n\left(h_{1}+h_{2}\right)}-e^{-ik_{0}n\left(h_{1}+h_{2}\right)}}{4\left(n+1\right)\sin\left(k_{0}nh_{1}\right)\sin\left(k_{0}nh_{2}\right)}+1\right\} \end{array}$$

Although Equation (14) is complicated, some simplified analyses can be made under certain conditions. It should be noted that according to Equation (13), transmission is reversely proportional to the square of *E_inc_*, so that a smaller absolute value of *E_inc_* will result in larger transmission. In Equation (14), the last term “1” in the brace represents the common Fresnel transmission. If the first two terms in the brace are small enough, then the PCR can be as high as common Fresnel transmission. It should be noted that there are sin(*k*_0_*nh*_1_) and sin(*k*_0_*nh*_2_) in both the first two terms in the brace. If any of them is 0, the first two terms will become infinitely large. Physically, sin(*k*_0_*nh*_1_) = 0/sin(*k*_0_*nh*_2_) = 0 means that the backward/forward propagating *y*-/*x*-polarized waves and the forward/backward propagating *y*-/*x*-polarized waves (reflected by the top/bottom metallic gratings) in the upper/lower dielectric separation interfere destructively with each other. Consequently, the plasmon resonances will be suppressed, and then the polarization will not be rotated. As a result, if *k*_0_*nh*_1_ or *k*_0_*nh*_2_ is an integer multiple of π, the PCR should be close to 0. To make the first two terms in the brace small enough, both sin(*k*_0_*nh*_1_) and sin(*k*_0_*nh*_2_) should be close to 1 or −1, which means that constructive interference happens. Then, the polarization conversion efficiency is promoted by enhanced plasmons, resulting in higher transmission. If *k*_0_*nh*_1_ and *k*_0_*nh*_2_ are both close to π/2, the second term in the brace is nearly 0. Then, the first term depends completely on *σ_u_*. Obviously, a large *σ_u_* will finally result in a small *E_inc_*, and then the transmission will be high. Here, *σ_u_* is expressed as [27,28]:

(15) $$\sigma_{u}\left(\omega \right)=\sigma_{u0}+\sum_{i=1}^{m}\sigma_{ui}$$

(16) $$\sigma_{v}\left(\omega \right)=0$$

where *σ_u_*_0_ is the Drude background conductivity:

(17) σu0ω=Diσ04EFπℏω+iτ

and *σ_ui_* corresponds to *i*th the resonance modes with:

(18) σuiω=iσ04βi2EFωπℏω2−ωi2+iωτ

Here, *σ*0 = *e*2/4*ℏ* is the universal conductivity of graphene, with *e* representing the electron charge and *ℏ* representing the reduced Plank constant. *βi*2 is the coupling strength between the incident light and the *i*th plasmon mode, *EF* is the Fermi level of the top graphene layer, *τ* is the relaxation time, *ω* is the angular frequency of the incidence, and *ωi* is the angular frequency of the *i*th resonance mode. *D* is a coefficient related to the duty cycle of graphene (the coverage of graphene in the *x*-*y* plane). In the absence of plasmon resonance and Drude background absorption in the *u* coordinate, both the amplitude and the phase of the incidence will not be changed by the plasmon-excitation layer, so that the polarization will not be rotated and the transmission will be 0. Correspondingly, *σu*(ω) = 0 and the first term in the brace of Equation (14) is infinite, resulting in 0 transmission. The polarization can only be rotated in the presence of the Drude background absorption or plasmon resonance.

## 3. Simulation Experiment Method

**Figure 2:**
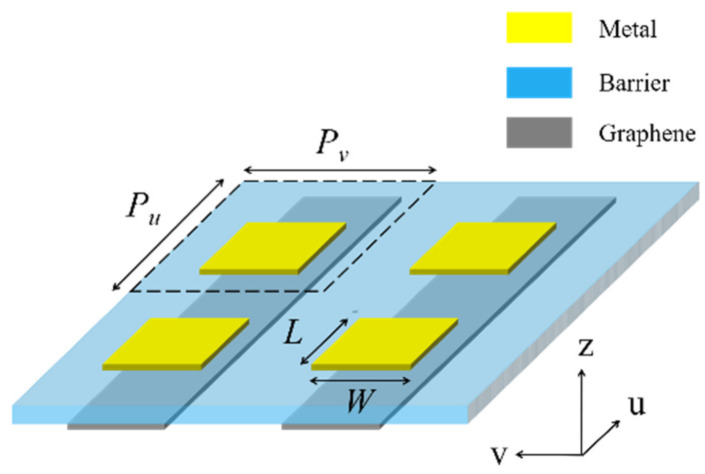
Graphene ribbons gated by metal cuboids (replacing the gray layer as shown in Figure 1).

To check the theoretical analyses, we performed FDTD simulations with Lumerical FDTD Solutions. As shown in Figure 2, we set gated graphene ribbons as the plasmon-excitation layer. The metal was modeled as a perfect electric conductor with 100 nm thickness. Graphene was modeled as an ultrathin ribbon with 0.5 nm thickness. The minimum meshes at the boundaries of graphene were 0.1 nm to promise the accuracy. Graphene was characterized by the Kubo formula [24,29]. The graphene ribbons extended along the *u* coordinate and the lengths of the metallic gates *L* in the *u* coordinate were initially set as 2.4 μm, so that gated modes could be excited in the *u* coordinate within the THz region. Graphene ribbons and metallic cuboids were separated by a dielectric barrier of 20 nm. The widths of the graphene ribbons and the metallic cuboids *W* along the *v* coordinate were fixed at 0.4 μm, so that the frequencies of the localized surface plasmon modes in the *v* coordinate were well beyond the THz region, and the Drude background absorption in the v coordinate is cancelled. The spacings between the graphene ribbons were fixed at 0.1 μm. *P_v_* and *P_u_* were initially set as 0.5 μm and 4 μm, respectively. Then, *D* in Equation (17) is 0.8 (0.4 μm width compared to *P_v_* = 0.5 μm). *n* was assumed to be 1.4, *h*_1_ and *h*_2_ were initially set as 14 μm, *E_F_* was initially set as 0.9 eV, and *τ* was 1 ps.

## 4. Results and Discussion

**Figure 3:**
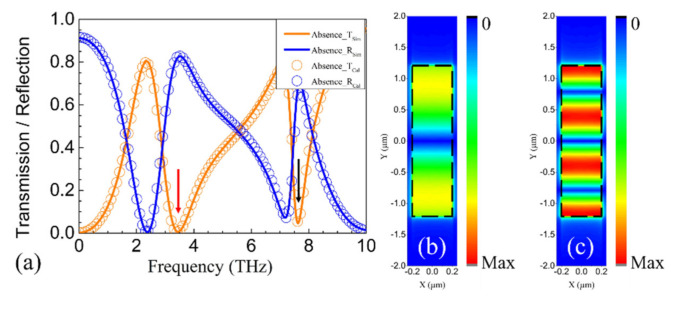

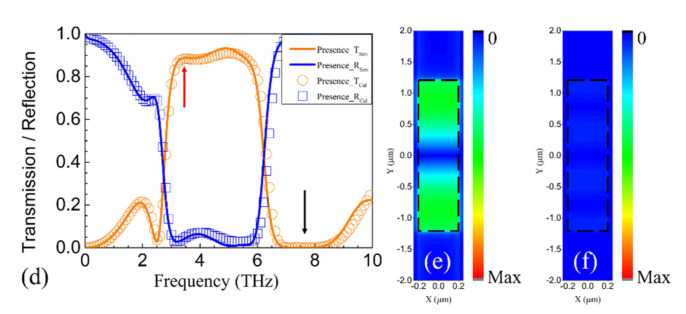
(**a**) The simulated (solid curves) and calculated (symbols) transmission (orange) and reflection (blue) spectra of the proposed structure in the absence of the metallic gratings. (**b**) The |*E_z_*| distributions at 3.42 THz (indicated by the red arrow in (**a**)) in the absence of the metallic gratings. (**c**) The |*E_z_*| distributions at 7.6 THz (indicated by the black arrow in (**a**)) in the absence of the metallic gratings. (**d**) The calculated and simulated transmission and reflection spectra of the proposed structure in the presence of the metallic gratings. (**e**) The |*E_z_*| distributions at 3.42 THz (indicated by the red arrow in (**d**)) in the presence of the metallic gratings. (**f**) The |*E_z_*| distributions at 7.6 THz (indicated by the black arrow in (**d**)) in the presence of the metallic gratings. The black dashed rectangles indicate the gated region.

The proposed structure was first simulated in the absence of the top and bottom metallic gratings. The resonance frequencies and the coupling strengths were retrieved by fitting the simulated spectra with the calculated ones. The frequencies of the first two resonance modes were found to be 3.42 and 7.6 THz, respectively, and the corresponding coupling strengths were 0.68 and 0.14, respectively. Figure 3a,d shows that the calculated and simulated spectra correspond well with each other, with or without the presence of the gratings, demonstrating the effectiveness of our model. In Figure 3d, PCR over 80% is realized between 3.04 and 5.91 THz. The corresponding relative bandwidth is 64.21%. Figure 3b,e shows the distribution of the absolute value of the z-component of the electric field |*E_z_*| between graphene and the metallic gratings at 3.42 THz, and Figure 3c,f shows the distribution of |*E_z_*| between graphene and the metallic gratings at 7.6 THz. It can be seen that dipolar and quadrupolar resonances are excited in the gated graphene region at 3.42 and 7.6 THz, respectively. Comparing Figure 3e,f, one can see that the quadrupolar resonance at 7.6 THz is extremely suppressed in the presence of the metallic gratings. It should be noted that *k*_0_*nh*_1_ = *k*_0_*nh*_2_ = 0.99π at 7.6 THz. As analyzed above, if *k*_0_*nh*_1_ and *k*_0_*nh*_2_ are close to integer multiples of π, plasmon resonances will be greatly suppressed by destructive interference. As a result, the polarization of the incidence is not rotated, and there is no transmission.

**Figure 4:**
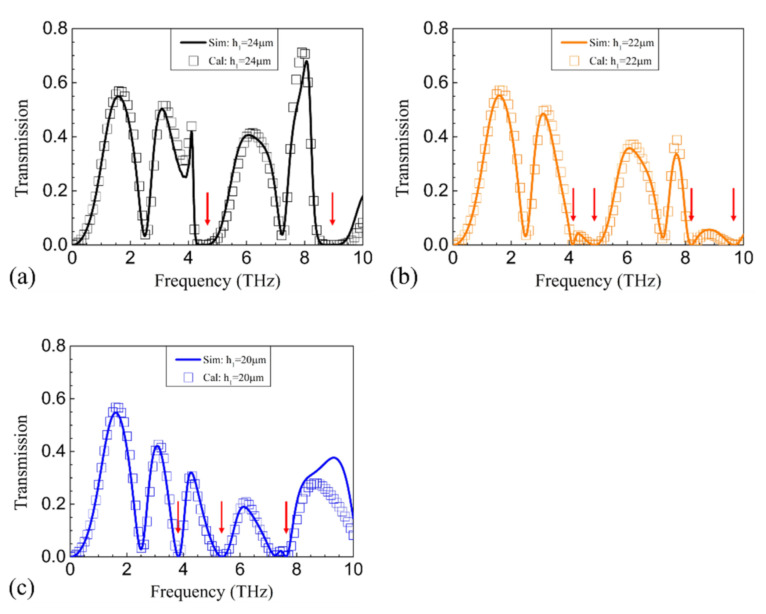
The simulated (solid curves) and calculated (symbols) transmission by reducing h_1_ from (**a**) 24 μm to (**b**) 22 μm and (**c**) 20 μm. The red arrows indicate the suppressed polarization conversion points due to the destructive interference.

To further show the effect of Fabry–Pérot-like cavities on the polarization conversion, *h*1 + *h*2 was fixed at 48 μm, with *h*1 decreased from 24 to 20 μm. As shown in Figure 4a, when *h*1 = 24 μm, the transmission at around 4.46 and 8.93 THz is nearly 0, where *k*0*nh*1 = *k*0*nh*2≈π and 2π, respectively. When *h*1 is reduced to 22 μm, the transmission at around 4.87, 4.12, 9.74, and 8.24 THz is nearly 0, where the former two frequencies correspond to *k*0*nh*1 = π and *k*0*nh*2 = π, respectively, and the latter two correspond to *k*0*nh*1 = 2π and *k*0*nh*2 = 2π, respectively. Similarly, when *h*1 is reduced to 20 μm, the transmission at around 5.34, 3.82, and 7.64 THz is nearly 0. It is obvious that the PCR between any two closely adjacent 0 transmission points are quite limited. Therefore, in order to realize broadband polarization conversion with high PCR, it would be better that the upper and lower dielectric separations share the same thickness.

**Figure 5:**
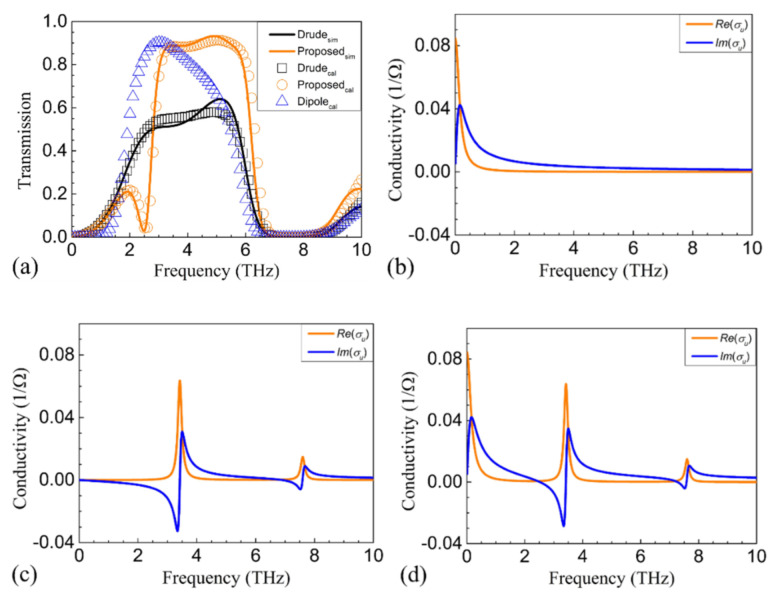
(**a**) The simulated transmission in the absence of metal squares (that is, the Drude background absorption-only case) (black solid curve), the simulated transmission of the proposed structure (orange solid curve), the calculated transmission in the absence of metal squares (black squares), the calculated transmission of the proposed structure (orange circles), and the calculated transmission of the dipolar absorption-only case (blue triangles). (**b**) The calculated *Re*(*σ_u_*) (orange) and *Im*(*σ_u_*) (blue) of the conductivity of Drude background absorption. (**c**) The calculated *Re*(*σ_u_*) (orange) and *Im*(*σ_u_*) (blue) of the conductivity of the dipolar resonance. (**d**) The calculated *Re*(*σ_u_*) (orange) and *Im*(*σ_u_*) (blue) of the proposed structure.

It may be questionable that the polarization of incidence at 5.9 THz can be rotated by dipolar resonance excited at 3.42 THz, while the polarization of incidence at 2.5 THz cannot be rotated. Here, we would like to stress that the dipolar resonance is not the only cause of the polarization conversion window. The Drude background absorption also contributes. In the absence of the metallic gratings, the proposed structure is similar to that in Zhao’s work [24], and Drude background absorption is the only contribution of the effective conductivity. Similarly, PCR over 25% can be observed between 2 and 6 THz, as shown in Figure 5a. In the model calculation, in the absence of the Drude background absorption, dipolar resonance will result in PCR over 25% between 1.75 and 6.1 THz, with a maximum PCR of about 90%, as shown by the blue triangles in Figure 5a. It seems strange that the relative bandwidths of both single absorptions are wider than their “cooperation”. However, it should be noted from Equation (18) that the imaginary part of the effective conductivity changes signs around resonance frequencies. As shown in Figure 5b,c, the Drude absorption results in a positive imaginary part of the effective conductivity, *Im*(*σ_u_*), while the dipolar resonance results in a negative *Im*(*σ_u_*) within 3.42 THz. For the real part of the effective conductivity, *Re*(*σ_u_*), the value is always close to 0 in the frequencies far away from a certain resonance mode. Thus, it can be concluded that *Re*(*σ_u_*) is the main contribution of the polarization conversion at a close vicinity to a resonance mode, while *Im*(*σ_u_*) is responsible for the broadening. Unfortunately, as shown in Figure 5d, the coexistence of the Drude background absorption and the dipolar resonance results in near-zero *Im*(*σ_u_*) at around 2.5 THz, which is harmful for the broadening at frequencies lower than 3.42 THz. For the frequencies higher than 3.42 THz, *Im*(*σ_u_*) is enhanced, which is beneficial for the broadening and PCR. Then, it can be concluded that the coexistence of Drude background absorption and excited plasmon modes will narrow the polarization conversion window at lower frequencies, while broadening the polarization conversion window at higher frequencies.

**Figure 6:**
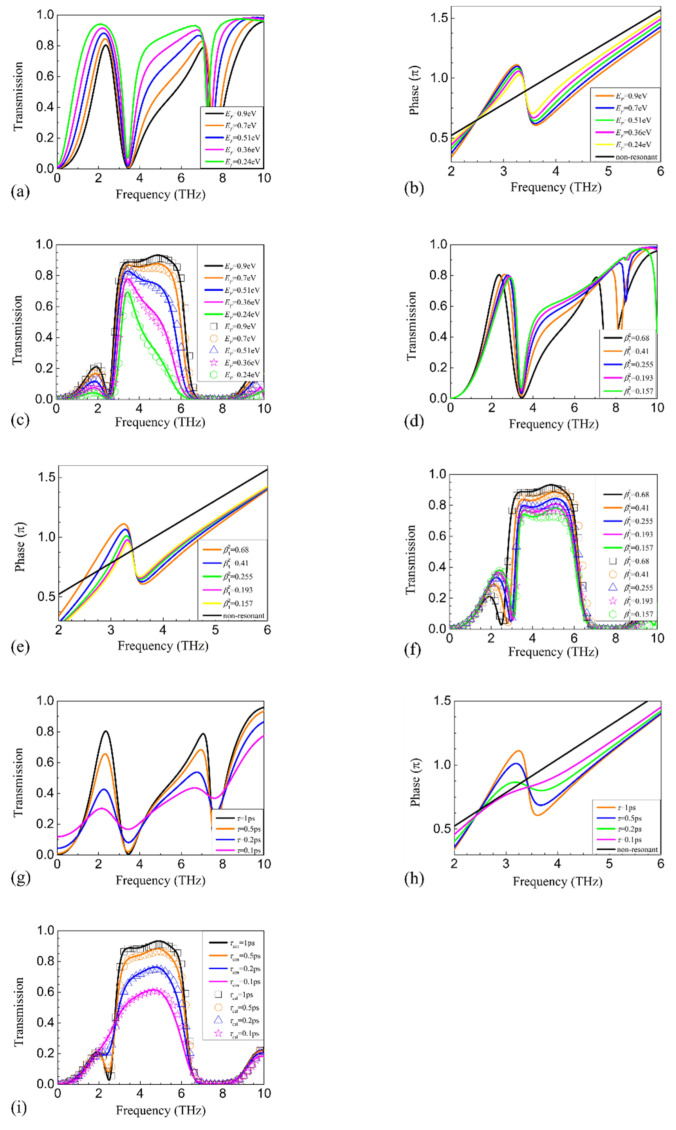
The simulated transmission spectra by decreasing (**a**) *E_F_*, (**d**) *β_i_*^2^, and (**g**) *τ*. The simulated change in phase by decreasing (**b**) *E_F_*, (**e**) *β_i_*^2^, and (**h**) *τ*. The simulated (solid curves) and calculated (symbols) transmission with the decrease in (**c**) *E_F_*, (**f**) *β_i_*^2^, and (**i**) *τ*.

Next, we consider the effect of *E_F_*, *β_i_*^2^, and *τ* on PCR. It can be anticipated from Equation (18) that a larger *E_F_*, *β_i_*^2^, and *τ* will result in larger *σ_ui_* and then higher transmission. Physically, larger *E_F_* and *β_i_*^2^ lead to more efficient coupling between the plasmons and the incidence radiation, and then more efficient modulation in the phase and amplitude of the incidence. Larger *τ* means less dissipative damping, less absorption, and hence stronger plasmon resonance, which is also beneficial for the modulation of the incidence. Then, we performed FDTD simulations in the absence of the metallic gratings. In order to keep the resonance frequencies and *β_i_*^2^ fixed when changing *E_F_*, *P_u_* was reduced to 3.5, 3.0, 2.5, and 2.5 μm, respectively. For each *P_u_*, the gate length was gradually reduced from 0.6*P_u_ to 0.2*P_u_. The corresponding simulated transmission spectra were fitted by theoretical calculation to retrieve the corresponding *β_i_*^2^. It should be pointed out that *E_F_* was fixed in the process while the resonance frequency was not. Once *β_i_*^2^ was found to be the same as the initial one, the gate length was picked, and then *E_F_* was changed to keep the resonance frequency the same as the initial one. For the above-mentioned *P_u_*, the gate lengths were reduced to 2.1, 1.8, 1.5, and 1.2 μm, respectively. The simulated spectra shown in Figure 6a show that the frequencies of the dipolar modes are fixed. Although not shown here, the calculation shows that the coupling strengths of the dipolar modes are also fixed. The modulation in the amplitude is weakened as *E_F_* reduces. Figure 6b shows that the modulation in the phase linearly decreases from ≈3.42 to 6 THz. As a result, both the PCR and the relative bandwidth greatly reduce from ≈3.42 to 6 THz, as shown in Figure 6c. In order to keep the resonance frequencies and *E_F_* fixed when changing *β_i_*^2^, *P_u_* was increased to 5.0, 6.0, 7.0, and 8.0 μm, respectively. Similar to the previous case, for each *P_u_*, the gate length was gradually reduced from 0.6*P_u_ to 0.1*P_u_ with *E_F_* fixed at 0.9 eV. Once the resonance frequency was the same as the initial one, *β_i_*^2^ was retrieved by fitting the calculated spectra with the theoretical calculation. Then, the gate length was reduced to 2.12, 1.98, 1.90, and 1.86 μm, respectively. We found in the calculation that *β*_1_^2^ reduces to 0.41, 0.255, 0.193, and 0.157, respectively. Although the modulation in amplitude and phase is also weakened as *β*_1_^2^ reduces, as shown in Figure 6d,e, the change is less than that shown in Figure 6a,b. Therefore, the reduction in PCR and relative bandwidth, as shown in Figure 6f is much less than that shown in Figure 6c. It may be questionable that both *E_F_* and *β*_1_^2^ are parts of the numerator in Equation (18), while the effects of *E_F_* and *β*_1_^2^ are different. Here, it should be pointed out that the difference comes mainly from the Drude background absorption. Comparing Figure 5b,d, one can see that the Drude background absorption also contributes a lot to the conductivity and then the modulation in amplitude and phase. A smaller *E_F_* will result in the weakening of the Drude background absorption, while a smaller *β*_1_^2^ will not, which causes the difference. As *τ* reduces from 1 to 0.1 ps, the transmission dips become broader, as shown in Figure 6g, so that the relative bandwidth as shown in Figure 6i is broadened. As shown in Figure 6h, the phase modulation reduces, especially around 3.42 THz. Consequently, the height of the polarization conversion window reduces, while the width broadens.

**Figure 7:**
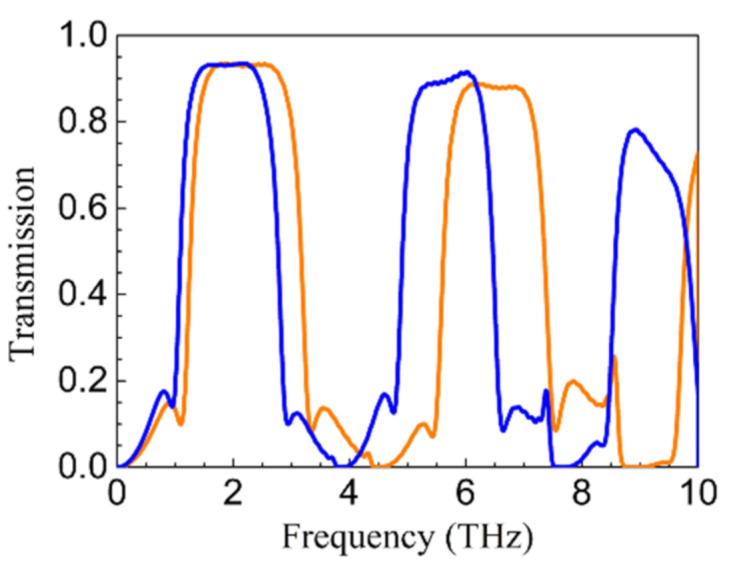
The transmission spectra for (*L*, *P_u_*, *h*_1_, *h*_2_) = (5.4, 6, 24, and 24 μm) (orange) and (6.4, 7, 28, and 28 μm) (blue).

It is shown in the above results that (i) PCR can be high in the vicinity of *k*0*nh*1 = *k*0*nh*2 = (2*k* + 1)π/2 (with a frequency ratio of 1:3:5:…) while being extremely low in the vicinity of *k*0*nh*1 = *k*0*nh*2 = *k*π with k as an arbitrary integer, and (ii) a larger *EF*, *βi*2, and *τ* will result in a higher and wider polarization conversion window. Note that the dispersion of gated graphene plasmons is linear instead of quadratic [30], and gated modes with a frequency ratio of 1:2:3:… will be efficiently excited in split-gate structures [31,32]. With this in mind, the efficiently excited odd-order gated modes can result in multi-broadband polarization conversion in the THz region. Now, (*L*, *P_u_*, *h*_1_, *h*_2_) are changed as (5.4, 6, 24, and 24 μm) and (6.4, 7, 28, and 28 μm), respectively. The simulated transmission shown in Figure 7 shows that two polarization conversion windows centered at 2.22 and 6.49 THz with PCR > 50% and a peak value of about 90% are obtained for the former, and three polarization conversion windows centered at 1.94, 5.69, and 9.2 THz are obtained for the latter. It can be inferred that more polarization conversion windows can be obtained in the THz frequencies if *L*, *P_u_*, *h*_1_ and *h*_2_ are further increased.

It should be pointed out that due to the co-existence of the top and bottom metallic gratings, the proposed structure can only be used to switch a linearly polarized incident beam into another linearly polarized beam. If either the top or the bottom gratings are replaced by non-tunable metamaterials, e.g., dielectrics or metals, or by tunable metamaterials, e.g., graphene, the incidence or the transmission can be elliptical or circular. Thus, future steps may include (i) changing the polarization of the transmission to ambient linear polarization by mechanically changing the direction of the metallic gratings; (ii) replacing the top or the bottom gratings with patterned graphene and changing the polarization of the transmission by electrically tuning the Fermi energy of graphene.

## 5. Conclusions

In conclusion, we applied the transfer matrix method, together with the harmonic oscillator model, to study the polarization conversion of a metallic grating/plasmon-excitation layer/metallic grating structure. The FDTD simulation and the model calculation results show that the constructive/destructive interaction between the forward and the backward waves in the dielectric spacings are beneficial/harmful for the excitation of plasmon resonances, thus enhancing/weakening the PCR. Both Drude absorption and the plasmon resonances contribute to the polarization conversion. Counteraction and cooperation on the imaginary part of the effective conductivity between the Drude background absorption and the plasmon resonances exist in lower frequencies and higher frequencies, respectively, so that the polarization conversion window is a little narrower, but with a higher PCR. A larger *E_F_*, *β_i_*^2^, and *τ* will result in a higher and wider polarization conversion window. Taking advantage of the linear dispersion relation of gated plasmons and the efficient excitation of the plasmons in split-gate structures, multi-broadband with PCR > 80% cross-polarization and active tunability is realized in the THz region. This study is helpful for the design of polarization rotators with similar sandwich structures, and it is beneficial for THz manipulation applications.

## Data Availability

The data presented in this study are available on request from the corresponding author.
